# Impaired Differentiation of Highly Proliferative ICOS^+^-Tregs Is Involved in the Transition from Low to High Disease Activity in Systemic Lupus Erythematosus (SLE) Patients

**DOI:** 10.3390/ijms22179501

**Published:** 2021-08-31

**Authors:** Florian Kälble, Lisa Wu, Hanns-Martin Lorenz, Martin Zeier, Matthias Schaier, Andrea Steinborn

**Affiliations:** 1 Department of Nephrology, University of Heidelberg, 69120 Heidelberg, Germany; Florian.Kaelble@med.uni-heidelberg.de (F.K.); Martin.Zeier@med.uni-heidelberg.de (M.Z.); matthias.schaier@med.uni-heidelberg.de (M.S.); 2 Department of Obstetrics and Gynecology, University of Heidelberg, 69120 Heidelberg, Germany; Lisa.Wu@med.uni-heidelberg.de; 3 Department of Rheumatology, University of Heidelberg, 69120 Heidelberg, Germany; Hanns-Martin.Lorenz@med.uni-heidelberg.de

**Keywords:** systemic lupus erythematosus (SLE), active disease, inducible costimulatory molecule (ICOS), regulatory T-cells (Tregs), recent thymic emigrants (RTEs), resting mature naïve cells (MNs), Treg/Tresp cell differentiation

## Abstract

Dysregulations in the differentiation of CD4^+^-regulatory-T-cells (Tregs) and CD4^+^-responder-T-cells (Tresps) are involved in the development of active systemic lupus erythematosus (SLE). Three differentiation pathways of highly proliferative inducible costimulatory molecule (ICOS)^+^- and less proliferative ICOS^−^-CD45RA^+^CD31^+^-recent-thymic-emigrant (RTE)-Tregs/Tresps via CD45RA^−^CD31^+^-memory-Tregs/Tresps (CD31^+^-memory-Tregs/Tresps), their direct proliferation via CD45RA^+^CD31^−^-mature naïve (MN)-Tregs/Tresps, and the production and differentiation of resting MN-Tregs/Tresp into CD45RA^−^CD31^−^-memory-Tregs/Tresps (CD31^−^-memory-Tregs/Tresps) were examined in 115 healthy controls, 96 SLE remission patients, and 20 active disease patients using six color flow cytometric analysis. In healthy controls an appropriate sequence of these pathways ensured regular age-dependent differentiation. In SLE patients, an age-independently exaggerated differentiation was observed for all Treg/Tresp subsets, where the increased conversion of resting MN-Tregs/Tresps particularly guaranteed the significantly increased ratios of ICOS^+^-Tregs/ICOS^+^-Tresps and ICOS^−^-Tregs/ICOS^−^-Tresps during remission. Changes in the differentiation of resting ICOS^+^-MN-Tresps and ICOS^−^-MN-Tregs from conversion to proliferation caused a significant shift in the ratio of ICOS^+^-Tregs/ICOS^+^-Tresps in favor of ICOS^+^-Tresps and a further increase in the ratio of ICOS^−^-Tregs/ICOS^−^-Tresps with active disease. The differentiation of ICOS^+^-RTE-Tregs/Tresps seems to be crucial for keeping patients in remission, where their limited production of proliferating resting MN-Tregs may be responsible for the occurrence of active disease flares.

## 1. Introduction

Systemic lupus erythematosus (SLE) is a chronic autoimmune disease characterized by periods of active disease and remission. The pathophysiology includes strong hyperactivity of B- and T-cells, resulting in autoantibody production against nuclear self-antigens and tissue injury in multiple organs, with lupus nephritis being the most common cause of morbidity and mortality [1,2,3,4,5]. Dysregulations in the differentiation of CD4^+^-T-cells seem to be the main cause of the disease, where it is still unclear which special T-helper cell subsets are involved. Historically, the Th1/Th2 balance is reckoned as decisively important [6]. Other subsets, such as inflammatory Th17 cells that promote inflammation [7,8,9] and follicular T-helper (Tfh) cells that enable autoantibody production, are recognized as key contributors to disease progression [10,11,12]. In addition, defects in regulatory T-cells (Tregs) are thought to provoke misdirected immune responses [13].

Initially, a general imbalance between Tregs and Tresps was presumed to cause disturbed CD4^+^-T-cell signaling in active disease patients [14]. In contrast, recent data from our own group revealed a significant decrease of total CD4^+^-T-helper cells in SLE patients, but indicated a shift in the composition of the total CD4^+^-T-helper cell pool in favor of Tregs. This shift was significantly more pronounced in patients with active disease compared to those in remission [15]. Other studies confirm these data not only for SLE but also for other autoimmune diseases such as rheumatoid arthritis and multiple sclerosis [16,17,18]. Such findings suggest that, in addition to quantitative discrepancies in the Treg/Tresp ratio, functional deficits regarding the suppressive capacity of the Tregs or an increased resistance of the Tresps to Treg suppression could play a role in the pathogenesis of SLE [19]. Meanwhile, there is evidence for a decisive role of both Treg and Tresp dysfunctions in these patients. Thus, it seems that disturbed Treg/Tresp cell differentiation causes both quantitative and functional deficits of both T-cell populations. We showed that impaired Treg suppressive activity in patients with SLE was due to altered Tresp cell differentiation, as well as to an unspecific immunosuppressive therapy affecting Treg cell differentiation [15]. However, which Treg/Tresp subsets are involved and to what extent the individual subsets are affected remains elusive. Moreover, the precise mechanisms of altered Treg/Tresp differentiation are not yet understood in detail.

We recently demonstrated that the thymic release of CD45RA^+^CD31^+^-recent thymic emigrant (RTE)-Tregs/Tresps may be disturbed under special pathological conditions such as renal insufficiency, forcing the already distributed RTE-Tregs/Tresp to differentiate more intensely into CD45RA^−^CD31^−^-memory Tregs/Tresps (CD31^−^-memory-Tregs/Tresps). Thus, the reinforcement of individual pathways via CD45RA^−^CD31^+^-memory-Tregs/Tresps (CD31^+^-memory-Tregs/Tresps) or resting CD45RA^+^CD31^−^-mature naïve (MN)-Tregs/Tresps influenced both functional properties of Tregs/Tresps, as well as quantitative changes concerning their ratio within total CD4^+^-T cells [20]. Since SLE is known to be associated with a highly accelerated activation and differentiation of the entire CD4^+^-T cell system we investigated which differentiation pathways of Tregs or Tresps are used during normal aging in healthy controls compared to SLE patients in remission or in patients with active disease. Moreover, we examined which pathways are age-independently strengthened and therefore probably become exhausted during active disease, as well as which ones are successfully suppressed by immunosuppressive therapy. Here, we distinguished between inducible costimulatory molecule (ICOS)^+^- and ICOS^−^-Tregs/Tresps, since our previous studies already showed that an accumulation of naïve cells occurred within total ICOS^+^-Tregs, indicating that immune senescence of these cells may play a central role in the development of active disease [21]. Thereby, ICOS-signaling equips ICOS^+^-Tregs and, presumably, ICOS^+^-Tresps with increased proliferation and survival abilities and therefore identifies both ICOS^+^-Tregs/Tresps as outstanding T-helper cell populations that may potentially affect the regulation of immune responses during autoimmune diseases [21,22]. Moreover, both T-cell subsets are localized in the human thymus and therefore may be released as independent naturally occurring RTE-Tregs/Tresps [23,24,25,26].

In this present study, we show that different pathways ensure adequate differentiation of these Treg/Tresp subsets with age. In SLE patients with active diseases, the individual pathways exhaust independent of age, while others still proceed and allow age-dependent differentiation. An increased differentiation via resting MN-Tregs/Tresps was ascertained for all Treg/Tresp subsets in active disease. Thus, resting ICOS^+^-MN Tregs converted while resting ICOS^+^-MN Tresps proliferated into CD31^−^-memory-Tregs/Tresps, causing a shift in the ratio of ICOS^+^-Tregs/ICOS^+^-Tresps in favor of ICOS^+^-Tresps. In contrast, converting resting ICOS^−^-MN Tregs changed to proliferation while resting ICOS^−^-MN-Tresps still converted, causing a shift in the ratio of ICOS^−^-Tregs/ICOS^−^-Tresps in favor of ICOS^−^-Tregs.

## 2. Results

### 2.1. CD4^+^-Lymphocytes of SLE Patients with Active Disease Show Signs of Premature Immune Senescence 

**Figure 1:**
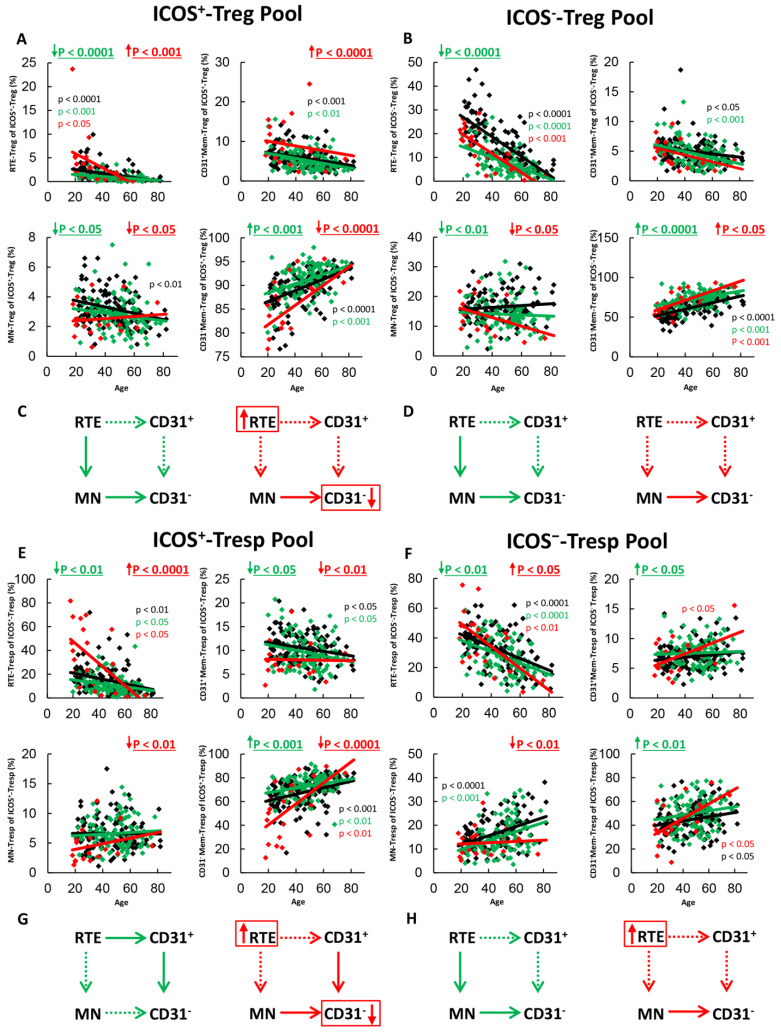
Changes in the composition of the total ICOS^+^- and ICOS^−^-Treg/Tresp pools with age in healthy volunteers, SLE patients in remission and SLE patients with active disease. The percentage of RTEs, MNs, CD31^+^-memory cells and CD31^−^-memory cells is shown for the ICOS^+^-Treg Pool (**A**), the ICOS^−^-Treg pool (**B**), the ICOS^+^-Tresp pool (**E**) and the ICOS^−^-Tresp pool (**F**) in healthy volunteers (Black coloured), SLE patients in remission (Green coloured) and in SLE patients with active disease (Red coloured). The figures present the regression lines concerning the changes in the percentages of the individual Treg/Tresp cell subsets with age. Significant age-dependent changes are marked by black, green, or red P-values. Significant age-independent differences between healthy volunteers and SLE patients in remission or SLE patients with active disease are marked by green or red underlined P-values. Age-independent increased differentiation of ICOS^+^ and ICOS^−^- RTE-Tregs/Tresps into CD31^−^-memory Tregs/Tresps via MN-Tregs/Tresps or CD31^+^-memory Tregs/Tresp is shown for SLE patients in remission (green arrows) and for SLE patients with active disease (red arrows) (**C**,**D**,**G**,**H**). Further information of the calculated P-values is illustrated in Table S1.

To show differences in the differentiation of ICOS^+^- and ICOS^−^-Tregs/Tresps between healthy volunteers (Group 1) and SLE patients in remission (Group 2) or SLE patients with a flare of the disease (Group 3), we determined the composition of the total CD4^+^-ICOS^+^- and ICOS^−^-Treg/Tresp pools with RTE-, MN-, CD31^+^- and CD31^−^-memory-cells in both healthy and affected individuals of different ages (Figure 1A,B,E,F). When recruited consecutively, 80% of patients in both the remission and the active disease group were found to be female, but only 20% were male. Therefore, we recruited an age-matched healthy control group with equal gender composition (Table 1).

In SLE remission patients, we found significantly decreased percentages of RTE-Tregs and MN-Tregs, but increased percentages of CD31^−^-memory-Tregs within both total ICOS^+^- and ICOS^−^-Tregs compared to healthy controls (Figure 1A,B), proposing an age-independent increased differentiation of both ICOS^+^- and ICOS^−^-RTE-Tregs via resting MN-Tregs into CD31^−^-memory-Tregs in these patients (Figure 1C,D). The ICOS^+^-Tresp pool contained significantly decreased percentages of RTE-Tresps and CD31^+^-memory-Tresps, but increased percentages of CD31^−^-memory-Tresps (Figure 1E), suggesting an age-independent increased differentiation of ICOS^+^-RTE-Tresps via CD31^+^-memory-Tresps into CD31^−^-memory-Tresps (Figure 1G). The total ICOS^−^-Tresp pool comprised significantly decreased percentages of RTE-Tresps, increased percentages of CD31^+^-memory-Tresps and increased percentages of CD31^−^-memory-Tresps in these patients (Figure 1F), proposing an inhibited differentiation via CD31^+^-memory Tresps, but increased differentiation via resting MN-Tresps (Figure 1H). Therefore, it seems that despite the immunosuppressive therapy, an increased ICOS^+^-Tresp cell differentiation via CD31^+^-memory-Tresps (Figure 1G) as well as an increased ICOS^−^-Tresp cell differentiation via resting MN-Tresps (Figure 1H) still occurs in SLE remission patients, which is counteracted by the increased differentiation of both ICOS^+^ and ICOS^−^-RTE-Tregs via resting MN-Tregs. 

In active disease patients, RTE-Tregs and CD31^+^-memory Tregs accumulated strongly, while MN-Tregs and CD31^−^-memory-Tregs decreased significantly within total ICOS^+^-Tregs (Figure 1A), proposing a strongly inhibited differentiation via CD31^+^-memory-Tregs which cannot be fully replaced by the increased differentiation of resting MN-Tregs into CD31^−^-memory-Tregs (Figure 1C). Within total ICOS^−^-Tregs, there was an increased differentiation of resting MN-Tregs into CD31^−^-memory Tregs (Figure 1B). Therefore, a certain degree of immune exhaustion can also be assumed for ICOS^−^-RTE-Tregs; however, it appears to be more pronounced for ICOS^+^-Tregs than for ICOS^−^-Tregs as a definite enrichment of RTEs could only be detected in the ICOS^+^-Treg pool (Figure 1C,D). Similarly, to ICOS^+^-Tregs, ICOS^+^-RTE-Tresps also accumulated within total ICOS^+^-Tresps, while all other subsets decreased significantly (Figure 1E). Hence, an accelerated differentiation of ICOS^+^-RTE-Tresps via both CD31^+^-memory-Tresps and resting-MN-Tresps seems to occur in these patients, which also exceeds its maximum capacity (Figure 1G). For ICOS^−^-Tresps, significantly increased percentages of RTE-Tresps, but decreased percentages of resting-MN-Tresps, were ascertained while the percentages of both CD31^+^- and CD31^−^-memory-Tresps remained unchanged (Figure 1F). Therefore, an exhaustion of ICOS^−^-RTE-Tresp differentiation via CD31^+^-memory Tresps, but still functioning differentiation of resting MN-Tresps may be assumed in active disease patients (Figure 1H). 

Overall, it is noticeable that the normal significant age-dependent differentiation of RTE-Tregs/Tresps into CD31^−^-Memory Tregs/Tresps in healthy controls is maintained in SLE remission patients and even in patients with active disease (Figure 1A,B,E,F). Only for ICOS^+^-Tregs, a significant age-dependent increase of CD31^−^-memory-Tregs was not ascertained (Figure 1A). Therefore, these data suggest that in SLE patients with active disease, certain differentiation pathways of RTE Tregs/Tresps may be enhanced and thus become exhausted, while others still occur and ensure age-related differentiation. Thereby, the additional differentiation of resting MN-Tregs/Tresps seems to be of special importance to achieve an increased differentiation.

### 2.2. ICOS^+^-RTE-Tregs of SLE Patients Show an Increased Differentiation via Converting Resting MN-Tregs which Exhausts in Active Disease

To identify the individual differentiation pathways of healthy controls and SLE patients in remission or active disease, we estimated the percentages of RTE-Tregs/Tresps within the total naïve Treg/Tresp pool during course of life. We correlated these percentages with the percentages of Ki67^+^ cells within total RTE-Tregs/Tresps, MN-Tregs/Tresps, CD31^+^-memory-Tregs/Tresps and CD31^−^-memory-Tregs/Tresps to see which pathways were strengthened with age, or in case of SLE, were intensified or already exhausted regardless of age. 

**Figure 2:**
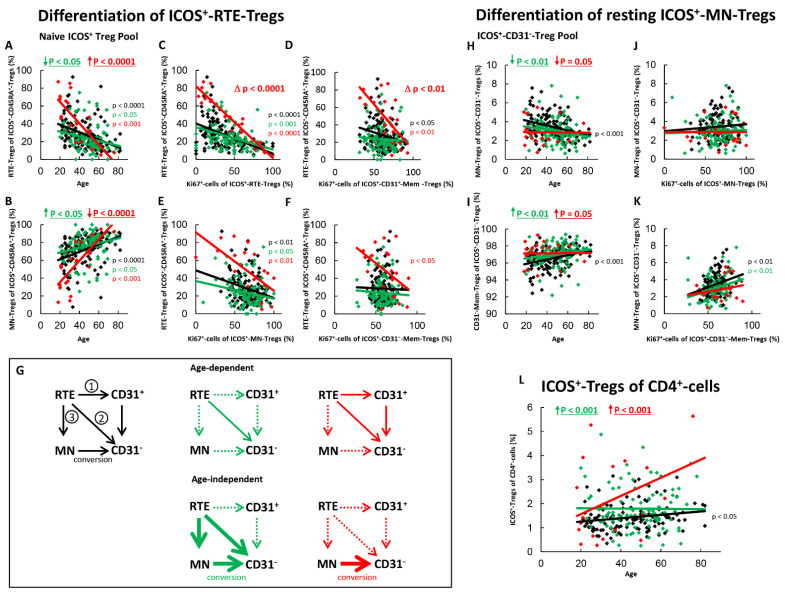
Differentiation of ICOS^+^-RTE-Tregs and resting ICOS^+^-MN-Tregs with age in healthy controls, SLE patients in remission and SLE patients with active disease. The percentages of RTE-Tregs and MN-Tregs within the naïve ICOS^+^-CD45RA^+^-Treg pool (**A**,**B**) as well as the percentages of MN-Tregs and CD31^−^-memory Tregs within the ICOS^+^-CD31^−^- Treg pool (**H**,**I**) were estimated depending on age in healthy volunteers (Black coloured), SLE patients in remission (Green coloured) and SLE patients with active disease (Red coloured). In order to examine the differentiation of ICOS^+^-RTE-Tregs, the percentage of RTE-Tregs within total naïve CD45RA^+^-Tregs was correlated with the percentage of Ki67^+^ cells within total RTE-Tregs (**C**), CD31^+^-memory-Tregs (**D**), MN-Tregs (**E**) and CD31^−^-memory-Tregs (**F**). In order to examine the differentiation of resting MN-Tregs, the percentage of MN-Tregs within total CD31^−^-Tregs was correlated with the percentage of Ki67^+^ cells within total MN-Tregs (**J**) and within total CD31^−^-memory-Tregs (**K**). Significant linear regression analyses are marked by black, green, or red P-values. Significant differences in the regression lines between healthy controls and SLE patients in remission or active disease are marked by green or red ∆ p values. Age-independent significant differences between healthy volunteers and study groups are marked by an arrow (↑↓) and their color-matched P-values. The arrow diagrams (**G**) schematize an increased (thick arrow), consistent (thin arrow) or decreased (dashed arrow) differentiation, color matched for the different patient groups. The resulting age-dependent and age-independent changes in the percentage of ICOS^+^-Tregs within total CD4^+^-T-helper cells are given in (**L**). Further information of the calculated P-values is illustrated in Table S1.

Figure 2 shows the results of these measurements concerning the differentiation of ICOS^+^-RTE-Tregs and resting ICOS^+^-MN-Tregs. For healthy controls, we found significantly decreasing percentages of RTE-Tregs and complementary increasing percentages of MN-Tregs within the naïve ICOS^+^-Treg pool with age (Figure 2A,B) and ascertained a significant negative correlation between the percentage of RTE-Tregs within naïve Tregs and the percentage of Ki67^+^ cells within total RTE-Tregs (Figure 2C). These data indicate a significant activation of ICOS^+^-RTE-Tregs with decreasing percentages of RTE-Tregs within their naive Treg pool (Figure 2C). As there was a similar significant negative correlation between the percentage of RTE-Tregs within naïve Tregs and the percentage of Ki67^+^ cells within CD31^+^-memory-Tregs (Figure 2D) and MN-Tregs (Figure 2E), it seems that with age, ICOS^+^-RTE-Tregs are activated and differentiate into CD31^+^-memory-Tregs, which subsequently proliferate into CD31^−^-memory-Tregs (Figure 2G, pathway 1), but also proliferate directly via MN-Tregs into CD31^−^-memory-Tregs (Figure 2G, pathway 2). A significant correlation between the percentage of RTE-Tregs and the percentage of Ki67^+^ cells within CD31^−^-memory-Tregs was not found (Figure 2F), indicating that with age, an additional differentiation pathway must exist which compensates the increasing percentage of Ki67^+^ cells within the CD31^−^-memory pool, caused by the increasing proliferation of RTE-Tregs and CD31^+^-memory-Tregs. To examine whether the differentiation of resting MN-Tregs could perform this task, we determined the percentages of MN-Tregs within total ICOS^+^-CD31^−^-Tregs and correlated its percentage with the percentage of Ki67^+^ cells within total MN-Tregs and within total CD31^−^-memory-Tregs (Figure 2H,K). We found significantly decreasing percentages of MN-Tregs and complementary increasing percentages of CD31^−^-memory-Tregs with age, indicating that resting-MN-Tregs additionally differentiate into CD31^−^-memory-Tregs (Figure 2H,I). We could not detect a significant correlation between the percentage of MN-Tregs within total CD31^−^-Tregs and their percentage of Ki67^+^ cells, proposing that RTE-Tregs, after proliferating into resting MN-Tregs, differentiate immediately into CD31^−^-memory-Tregs, without any activation induced regulation (Figure 2J, pathway 3). As there was a significant positive correlation between the percentage of MN-Tregs within total CD31^−^-Tregs and the percentage of Ki67^+^ cells within CD31^−^-memory-Tregs (Figure 2K), it can be assumed that the differentiation of resting MN-Tregs, presumably by their conversion, reduces the proliferation capacity of CD31^−^-memory-Tregs, keeping them susceptible to further activation (Figure 2G, pathway 3). In summary, it seems that the increased age-dependent differentiation of IOCS^+^-RTE-Tregs via all three pathways causes a significant increase of total ICOS^+^-Tregs within total CD4^+^-helper cells with age (Figure 2L).

For SLE patients in remission, an age-independent significant shift in the composition of the naïve ICOS^+^-Treg pool in favor of MN-Tregs was observed (Figure 2A,B). This means that there is an age-independent increased differentiation of RTE-Tregs in these patients via pathway 1 or 2, or by the increased production of resting MN-Tregs (pathway 3). Since we could not detect a significant correlation between the percentage of RTE-Tregs within the naïve Treg pool and the percentage of Ki67^+^ cells with total CD31^+^-memory Tregs (Figure 2D), but one between the percentage of RTE-Tregs and the percentage of Ki67^+^ cells within total MN-Tregs (Figure 2E), it may be assumed that the differentiation of ICOS^+^-RTE-Tregs via CD31^+^-memory-Tregs (Figure 2G, pathway 1) may be suppressed in these patients, while their proliferation via resting MN-Tregs into CD31^−^-memory-Tregs (Figure 2G, pathway 2) still occurs. We also observed a strongly increased age-independent differentiation of resting-MN-Tregs into CD31^−^-memory-Tregs (Figure 2H,I), but did not detect any differences in their activation induced differentiation capacity (Figure 2J) or their conversion into CD31^−^-memory-Tregs (Figure 2K). This means that the increased production of resting MN-Tregs results in the immediate conversion of resting-MN-Tregs into CD31^−^-memory-Tregs (Figure 2G, pathway 3). Probably this leads to a significant age-independent increase in the proportion of ICOS^+^-Treg within the total CD4^+^-T-helper cell pool in SLE remission patients (Figure 2L), while with age, only direct proliferation of the RTE-Tregs via MN-Tregs was ascertained for these patients (Figure 2G, pathway 2). 

In SLE patients with active disease, a significant age-dependent decrease, but an age-independent accumulation of ICOS^+^-RTE-Tregs compared to healthy controls within the naïve ICOS^+^-Treg pool became apparent (Figure 2A,B). Such findings propose that one or more pathways are strongly enhanced and therefore become exhausted, independent of age, while others still occur and ensure increasing differentiation of RTE-Tregs with age. A significant negative correlation between the percentage of ICOS^+^-RTE-Tregs within the naïve ICOS^+^-Treg pool and the percentage of Ki67^+^ cells within RTE-Tregs, MN-Tregs, CD31^+^- and CD31^−^-memory-Tregs was ascertained for these patients indicating that the age-dependent activation of the RTE-Tregs and their differentiation via CD31^+^-memory-Tregs or via their direct proliferation into CD31^−^-memory-Tregs is maintained during active disease (Figure 2C–E) causing a significant increase in the proliferation capacity of the CD31^−^-memory pool with age (Figure 2F). However, a significant impairment in the overall differentiation capacity of the ICOS^+^-RTE-Tregs (Figure 2C), especially via pathway 1 (Figure 2D) was ascertained, as the slope of the regression lines were significantly different between healthy controls and patients with active disease. Therefore, it can be assumed that strong age-independent differentiation of ICOS^+^-RTE-Tregs via CD31^+^-memory-Tresps in younger patients causes exhaustion of this differentiation pathway with age (Figure 2G, pathway 1) while their direct proliferation is not significantly affected (Figure 2G, pathway 2). Regarding the differentiation via resting MN-Tregs, it is noticeable that although there was an age-independently increased differentiation of resting MN-Tregs into CD31^−^-memory-Tregs (Figure 2H,I), these presumably converted cells were not able to significantly reduce the proportion of Ki67^+^ cells within the CD31^−^-memory-Treg pool (Figure 2K). An age-dependent differentiation of resting MN-Tregs into CD31^−^-memory-Tregs was not observed in these patients (Figure 2H,I), presumably because their production also ceased with age (Figure 2G, pathway 3). Consequently, the significant increase in the proliferation capacity of the CD31^−^-memory-Tregs with age could not be counteracted (Figure 2F). In summary, these data propose that an age-independent exhaustion of differentiation pathways 1 and 3 provokes the age-independent accumulation of RTE-Tregs within the naïve ICOS^+^-Treg pool while mainly direct proliferation of the RTE-Tregs ensures their age-dependent differentiation (Figure 2G). Nevertheless, compared to healthy controls, the proportion of ICOS^+^-Treg within the total CD4^+^-T-helper cell pool was found to be significantly increased in these patients (Figure 2L). 

### 2.3. ICOS^+^-RTE-Tresps of SLE Patients Show an Increased Differentiation via Proliferating Resting MN-Tresps which Exhausts in Active Disease

**Figure 3:**
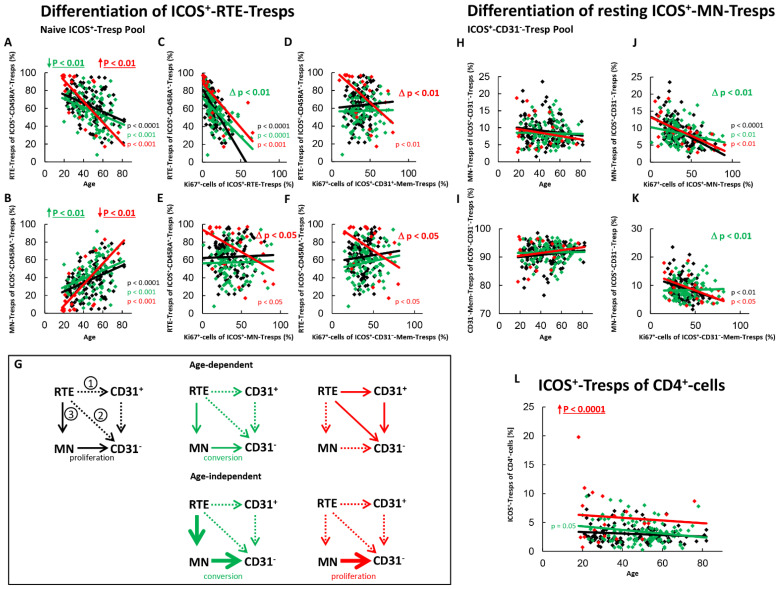
Differentiation of ICOS^+^-RTE-Tresps and resting ICOS^+^-MN-Tresps with age in healthy controls, SLE patients in remission and SLE patients with active disease. The percentages of RTE-Tresps and MN-Tresps within the naïve ICOS^+^-CD45RA^+^-Tresp pool (**A**,**B**) as well as the percentages of MN-Tresps and CD31^−^-memory Tresps within the ICOS^+^-CD31^−^- Tresp pool (**H**,**I**) were estimated depending on age, in healthy volunteers (Black coloured), SLE patients in remission (Green coloured) and SLE patients with active disease (Red coloured). In order to examine the differentiation of ICOS^+^-RTE-Tresps, the percentage of RTE-Tresps within total naïve CD45RA^+^-Tresps was correlated with the percentage of Ki67^+^ cells within total RTE-Tresps (**C**), CD31^+^-memory-Tresps (**D**), MN-Tresps (**E**) and CD31^−^-memory-Tresps (**F**). In order to examine the differentiation of resting MN-Tresp, the percentage of MN-Tresps within total CD31^−^-Tresps was correlated with the percentage of Ki67^+^ cells within total MN-Tresps (**J**) and within total CD31^−^-memory-Tresps (**K**). Significant linear regression analyses are marked by black, green, or red P-values. Significant differences in the regression lines between healthy controls and SLE patients in remission or active disease are marked by green or red ∆ p values. Age-independent significant differences between healthy volunteers and study groups are marked by an arrow (↑↓) and their color-matched P-values. The arrow diagrams (**G**) schematize an increased (thick arrow), consistent (thin arrow) or decreased (dashed arrow) differentiation, color matched for the different patient groups. The resulting age-dependent and age-independent changes in the percentage of ICOS^+^-Tresps within total CD4^+^-T-helper cells are given in (**L**). Further information of the calculated P-values is illustrated in Table S1.

Figure 3 shows the differentiation of ICOS^+^-RTE- and resting ICOS^+^-MN-Tresps of all three patient groups. For healthy controls, we found an age-dependent shift in the composition of the total naïve ICOS^+^-Tresp pool in favor of MN-Tresps (Figure 3A,B). A significant correlation between its percentage of RTE-Tresps and their percentage of Ki67^+^ cells indicated a significant activation of the RTE-Tresps with age (Figure 3C). However, a significant correlation between the percentage of RTE-Tresps and the percentage of Ki67^+^ cells within total MN-, or CD31^+^-memory-Tresps was not detectable (Figure 3D,E). Therefore, the ICOS^+^-RTE-Tresps do not seem to differentiate via CD31^+^-memory-Tresps (Figure 3G, pathway 1) or direct proliferation (Figure 3G, pathway 2) into CD31^−^-memory-Tresps, but rather differentiate via resting MN-Tresps into CD31^−^-memory Tresps. This is recognizable by the fact that the naive Tresp pool shifts significantly with age in favor of MN-Tresps (Figure 3A,B), whereas the CD31^−^-Tresp pool remains unchanged (Figure 3H,I). Since a significant correlation between the percentage of resting-MN-Tresps within total CD31^−^-Tresps and their percentage of Ki67^+^ cells within resting MN-Tresps was ascertained (Figure 3J), it must be assumed that resting MN-Tresps are activated in case of their decreased production and thereby forced to differentiate into CD31^−^-memory-Tresps. In healthy controls, such a differentiation of resting MN-Tresps seems to occur with age. However, in contrast to ICOS^+^-resting MN-Tregs, these cells do not appear to be converted into CD31^−^-memory Tresps, but rather proliferate into CD31^−^-memory-Tresps, since the proportion of Ki67^+^ cells within CD31^−^-memory-Tresps increases significantly with increasing differentiation of resting MN-Tresps into CD31^−^-memory Tresps (Figure 3K). Therefore, it seems that there is a significant age-dependent differentiation of RTE-Tresps into resting MN-Tresps (Figure 3G, pathway 3) which proliferate into CD31^−^-memory-Tresps in healthy controls. As the differentiation of ICOS^+^-RTE-Tresps is limited to pathway 3, an age-related increase of ICOS^+^-Tresps within total CD4^+^-T-helper cells could not be determined (Figure 3L). 

In SLE remission patients, both an age-dependent and an age-independent significant shift in the composition of the naïve ICOS^+^-Tresp pool in favor of MN-Tresps was observed (Figure 3A,B). This was accompanied by a significantly intensified activation of the RTE-Tresps (Figure 3C). However, an increased differentiation of the RTE-Tresps via CD31^+^-memory-Tresps (Figure 3D) or via direct proliferation (Figure 3E) was not ascertained. Instead, a significantly strengthened differentiation capacity of resting-MN-Tresps into CD31^−^-memory-Tresps was detected (Figure 3J). Since the percentage of resting-MN-Tresps was not age-independently increased within total CD31^−^-memory-Tresps, enhanced age-independent differentiation of resting-MN-Tresps seems to have occurred (Figure 3H,I). Furthermore, their proliferation into CD31^−^-memory-Tresps was also found to be significantly reduced and presumably changed to conversion (Figure 3K). Therefore, it seems that there may be an age-dependently, as well as an age-independently increased production of resting MN-Tresps in SLE remission patients, where their proliferation into CD31^−^-memory-Tresps was relatively effectively blocked (Figure 3G, pathway 3). Thus, ICOS^+^-Tresps decreased with age within total CD4^+^-T-helper cells and an age-independent increase of these cells seemed to be sustainably prevented in these patients (Figure 3L). 

In SLE patients with active disease, a significant age-dependent decrease, but age-independent accumulation of RTE-Tresps within the naïve ICOS^+^-Tresp pool, was ascertained. These findings suggest that like ICOS^+^-RTE-Tregs, certain pathways of these cells are age-independently enhanced and therefore become exhausted, while others are less affected and maintain age-dependent differentiation in these patients (Figure 3A,B). In contrast to ICOS^+^-RTE-Tregs, a significant impairment in the age-dependent activation of ICOS^+^-RTE-Tresps between healthy controls and patients with active disease was not observed (Figure 3C). Rather, it was noticed that ICOS^+^-RTE-Tresps of patients with active disease, in contrast to those of healthy controls, who showed neither an age-dependent differentiation via CD31^+^-memory Tresps nor a direct proliferation, used both pathways for their age-dependent production of CD31^−^-memory-Tresps (Figure 3D,E,G, pathway 1 and pathway 2). Like ICOS^+^-Tregs, this caused a significantly increasing proliferation capacity of the CD31^−^-memory-Tresps with age (Figure 3F). An additional age-dependent differentiation of resting MN-Tresps into CD31^−^-memory Tresps was not observed (Figure 3H,I). Since there was a significant age-independent shift in the naïve Tresp pool in favor of RTEs, but no age-independent decrease of resting MN-Tresps within total CD31^−^-Tresps, resting MN-Tresps seemed to have accumulated within CD31^−^-Tresps (Figure 3H,I), while their differentiation capacity decreased again (Figure 3J) so that the resting MN-Tresps again proliferated into CD31^−^-memory-Tresps (Figure 3K). In summary, our data propose that there is an enhanced age-independent proliferation of resting MN-Tresps into CD31^−^-memory-Tresps (Figure 3G, pathway 3). Since this pathway exhausts, particularly in young patients, differentiation of RTE-Tresps via CD31^+^-memory-Tresps or direct proliferation (Figure 3G, pathway 1 and 2) occurs mainly in elderly patients. Thereby, the increased proliferation (Figure 3G, pathway 3) seems to cause an age-independent increase of ICOS^+^-Tresp within total CD4^+^-T-helper cells in these patients (Figure 3L). 

### 2.4. ICOS^−^-RTE-Tregs of SLE Patients Show an Increased Differentiation via Converting Resting MN-Tregs which Changes to Proliferation and Exhausts in Active Disease

**Figure 4:**
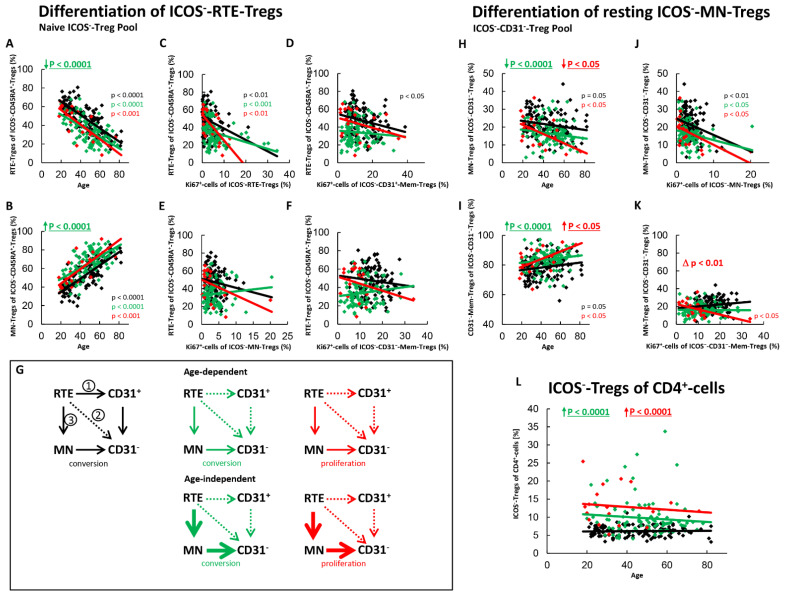
Differentiation of ICOS^−^-RTE-Tregs and resting ICOS^−^-MN-Tregs with age in healthy controls, SLE patients in remission and SLE patients with active disease. The percentages of RTE-Tregs and MN-Tregs within the naïve ICOS^−^-CD45RA^+^-Treg pool (**A**,**B**) as well as the percentages of MN-Tregs and CD31^−^-memory Tregs within the ICOS^−^-CD31^−^- Treg pool (**H**,**I**) were estimated depending on age, in healthy volunteers (Black coloured), SLE patients in remission (Green coloured) and SLE patients with active disease (Red coloured). In order to examine the differentiation of ICOS^−^-RTE-Tregs, the percentage of RTE-Tregs within total naïve CD45RA^+^-Tregs was correlated with the percentage of Ki67^+^ cells within total RTE-Tregs (**C**), CD31^+^-memory-Tregs (**D**), MN-Tregs (**E**) and CD31^−^-memory-Tregs (**F**). In order to examine the differentiation of resting MN-Tregs, the percentage of MN-Tregs within total CD31^−^-Tregs was correlated with the percentage of Ki67^+^ cells within total MN-Tregs (**J**) and within total CD31^−^-memory-Tregs (**K**). Significant linear regression analyses are marked by black, green, or red P-values. Significant differences in the regression lines between healthy controls and SLE patients in remission or active disease are marked by green or red ∆ p values. Age-independent significant differences between healthy volunteers and study groups are marked by an arrow (↑↓) and their color-matched P-values. The arrow diagrams (**G**) schematize an increased (thick arrow), consistent (thin arrow) or decreased (dashed arrow) differentiation, color matched for the different patient groups. The resulting age-dependent and age-independent changes in the percentage of ICOS^−^-Tregs within total CD4^+^-T-helper cells are given in (**L**). Further information of the calculated P-values is illustrated in Table S1.

Figure 4 shows the differentiation of ICOS^−^RTE-Tregs and resting ICOS^−^MN-Tregs for both SLE patients in remission and for those with active disease compared to healthy controls. In healthy controls, with age, a significant shift in the composition of the naïve Treg pool in favor of MN-Tregs was detected (Figure 4A,B). Accordingly, a significantly increased activation (Figure 4C), and a significantly increased differentiation of the RTE-Tregs via CD31^+^-memory-Tregs (Figure 4D,G, pathway 1) was ascertained. An increased proliferation of the RTE-Tregs with age (Figure 4E,G, pathway 2) was not observed. Additionally, resting MN-Tregs differentiated into CD31^−^-memory-Tregs (Figure 4H,I), if their production was too low (Figure 4J). This did not increase the proportion of Ki67^+^-cells within CD31^−^-memory-Tregs (Figure 4K), so it appears that these cells were largely converting rather than proliferating into CD31^−^-memory-Tregs (Figure 4G, pathway 3). The overall proliferation capacity of the CD31^−^-memory-Tregs did not change significantly with age (Figure 4F), obviously because the increased proliferation of the CD31^+^-memory-Tregs was sufficiently neutralized by this increased conversion of resting ICOS^−^MN-Tregs. Age-dependent changes of ICOS^−^-Tregs within CD4^+^-T helper cells were not observed (Figure 4L).

For SLE patients in remission, we revealed both an age-dependent as well as an age-independent significantly increased shift in the composition of the naïve ICOS^−^-Treg pool in favor of MN-Tregs (Figure 4A,B). Since we could not detect a significant correlation between the percentage of RTE-Tregs within the naïve Treg pool and the percentage of Ki67^+^ cells within total CD31^+^-memory Tregs or MN-Tregs, it can be assumed that the differentiation via CD31^+^-memory-Tregs or via direct proliferation of the RTE-Tregs does not occur in these patients (Figure 4D,E,G, pathway 1 and 2). Consequently, with age, RTE-Tregs differentiated only via resting MN-Tregs into CD31^−^-memory Tregs (Figure 4G, pathway 3). Additionally, an age-independent significantly increased differentiation of resting-MN-Tregs into CD31^−^-memory-Tregs was observed in these patients (Figure 4H,I). Their differentiation capacity was not significantly different from that of healthy controls (Figure 4J). Since the proportion of Ki67^+^ cells within CD31^−^-Tregs did not increase with increasing differentiation (Figure 4K), these cells also seemed to be predominantly converted into CD31^−^-memory-Tregs (Figure 4G, pathway 3). Obviously, this age-independently increased differentiation of RTE-Tregs via resting MN-Tregs into CD31^−^-memory-Tregs leads to an age-independently increased proportion of ICOS^−^-Tregs within total CD4^+^-T-helper cells in SLE remission patients (Figure 4L). 

In patients with active disease, an age-independent shift in the composition of the naïve ICOS^−^-Treg pool in favor of MN-Tregs was observed, however significance was not achieved (Figure 4A,B). On the other hand, both an age-dependent and an age-independent significantly increased differentiation of resting-MN-Tregs into CD31^−^-memory-Tregs was confirmed (Figure 4H,I). We did not detect a significant difference in the differentiation capacity of resting MN-Tregs between healthy controls and patients with active disease (Figure 4J). However, as the differentiation of resting MN-Tregs increased the percentage of Ki67^+^ cells within CD31^−^-memory-cells significantly, it seems that these cells proliferated into CD31^−^-memory-Tregs (Figure 4K). Therefore, a significant change in the differentiation of the resting-MN-Tregs from conversion to proliferation may occur during active disease (Figure 4K), indicating that resting MN-Tregs are not sufficiently produced in these patients (Figure 4G, pathway 3). With age, an increasing activation of the RTE-Tregs was observed (Figure 4C). However, an age-dependent differentiation via CD31^+^-memory-Tregs (Figure 4D,G, pathway 1) or direct proliferation (Figure 4E,G, pathway 2) did not occur. Instead, an increasing differentiation of RTE-Tregs via proliferating resting MN-Tregs was observed with age (Figure 4H,I). In summary, it seems that there is both an age-dependent and an age-independent exhaustion of the differentiation via 31^+^-memory-Tregs and resting MN-Tregs, causing a proliferation of resting-MN-Tregs in these patients (Figure 4G, pathway 3). Presumably, this increased differentiation via proliferating resting MN-Tregs causes a significant age-independent increase of ICOS^−^-Tregs within total CD4^+^-T-helper cells (Figure 4L). 

### 2.5. ICOS^−^-RTE-Tresps of SLE Patients Show an Increased Differentiation via Converting Resting MN-Tresps which Exhausts in Active Disease 

**Figure 5:**
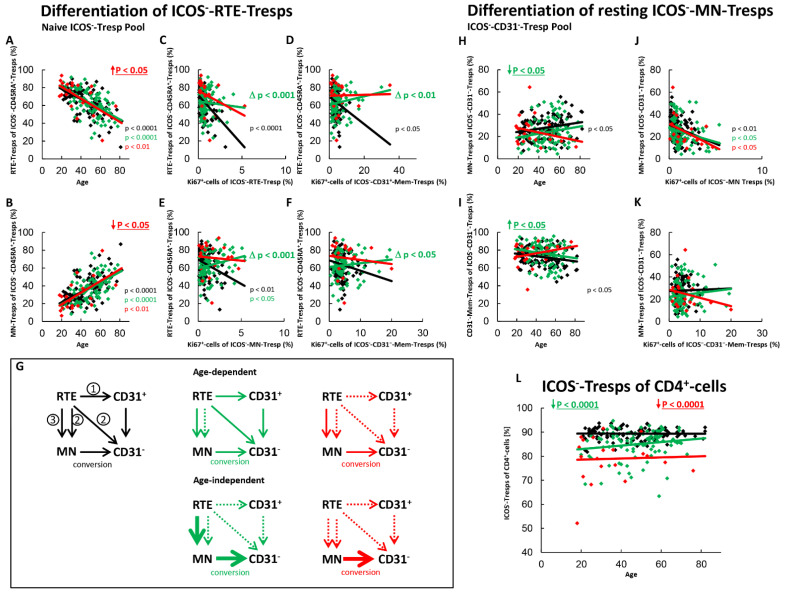
Differentiation of ICOS^−^-RTE-Tresps and resting ICOS^−^-MN-Tresps with age in healthy controls, SLE patients in remission and SLE patients with active disease. The percentages of RTE-Tresps and MN-Tresps within the naïve ICOS^−^-CD45RA^+^-Tresp pool (**A**,**B**) as well as the percentages of MN-Tresps and CD31^−^-memory Tresps within the ICOS^−^-CD31^−^- Tresp pool (**H**,**I**) were estimated depending on age, in healthy volunteers (Black coloured), SLE patients in remission (Green coloured) and SLE patients with active disease (Red coloured). In order to examine the differentiation of ICOS^−^-RTE-Tresps, the percentage of RTE-Tresps within total naïve CD45RA^+^-Tresps was correlated with the percentage of Ki67^+^ cells within total RTE-Tresps (**C**), CD31^+^-memory-Tresps (**D**), MN-Tresps (**E**) and CD31^−^-memory-Tresps (**F**). In order to examine the differentiation of resting MN-Tresp, the percentage of MN-Tresps within total CD31^−^-Tresps was correlated with the percentage of Ki67^+^ cells within total MN-Tresps (**J**) and within total CD31^−^-memory-Tresps (**K**). Significant linear regression analyses are marked by black, green, or red P-values. Significant differences in the regression lines between healthy controls and SLE patients in remission or active disease are marked by green or red ∆ p values. Age-independent significant differences between healthy volunteers and study groups are marked by an arrow (↑↓) and their color-matched P-values. The arrow diagrams (**G**) schematize an increased (thick arrow), consistent (thin arrow) or decreased (dashed arrow) differentiation, color matched for the different patient groups. The double arrow relates to the enrichment of resting MN-cells. The resulting age-dependent and age-independent changes in the percentage of ICOS^−^-Tresps within total CD4^+^-T-helper cells are given in (**L**). Further information of the calculated P-values is illustrated in Table S1.

Investigating the differentiation of ICOS^−^-RTE-Tresps and resting ICOS^−^-MN-Tresps (Figure 5) in healthy controls, we revealed that RTE-Tresps were also significantly activated with age (Figure 5A–C) and differentiated via CD31^+^-memory-Tresps (Figure 5D,G, pathway 1) or direct proliferation into CD31^−^-memory-Tresps (Figure 5E,G, pathway 2). An additional significant accumulation of resting MN-Tresps within CD31^−^-Tresps seems to occur in these patients (Figure 5H,I). Nevertheless, there was a significant activation-induced differentiation of resting-MN-Tresps into CD31^−^-memory-Tresps in healthy controls (Figure 5J). The increase of MN-Tresps within CD31^−^-Tresps may therefore be caused by insufficiently proliferating RTE-Tresps accumulating as resting MN-Tresps with age (Figure 5G, pathway 2). As there was no negative correlation between the percentage of resting MN-Tresps within CD31^−^-Tresps and the percentage of Ki67^+^-cells within CD31^−^-memory-Tresps the resting MN-Tresps seemed to be largely converted into CD31^−^-memory-Tresps (Figure 5K,G, pathway 3), keeping the proliferation capacity of the CD31^−^-memory-Tresps stable with age (Figure 5F).

For SLE patients in remission, an age-independent significant shift in the composition of the naïve ICOS^−^-Tresps in favor of MN-Tresps was not observed (Figure 5A,B), although an age-independent significantly increased conversion of resting MN-Tresps into CD31^−^-memory-Tresps was detected (Figure 5G,H–K, pathway 3). This means that an age-independent accumulation of RTE-Tresps must have been occurred in the naïve Tresp pool, which makes the age-independent decrease of RTEs in the naive pool undetectable (Figure 5A,B). An overall age-dependent activation of the RTE-Tresps could not be ascertained for these patients (Figure 5C). However, a positive correlation for the percentage of RTE-Tresps within total naïve Tresps and the percentage of Ki67^+^-cells within CD31^+^-memory Tresps and MN-Tresps was observed in these patients (Figure 5D,E). This means that age-dependent differentiation of RTE-Tresps occurs in these patients, even if RTE-Tresps accumulate within the naïve Tresp pool. However, this differentiation seems to take place to a much lesser extent compared to healthy volunteers. Thereby it seems that the RTE-Tresps differentiate via both CD31^+^-memory Tresps and via proliferating RTE-Tresps into CD31^−^-memory-Tresps (Figure 5G, pathway 1 and 2). In addition, similarly to healthy controls, an age-dependent conversion of resting MN-Tresps seems to occur in these patients (Figure 5H–K and Figure 5G, pathway 3). In summary, the decreased percentage of ICOS^−^-Tresps within CD4^+^-T-helper cells shows that although there is an age-independently increased differentiation of resting MN-Tresps into CD31^−^-memory-Tresps (Figure 5G, pathway 3), this differentiation does not compensate the less effective differentiation of RTE-Tresps via CD31^+^-memory-Tresps and via direct proliferation compared to healthy controls (Figure 5L). 

In SLE patients with active disease, an age-independent significant accumulation of RTE-Tresps within the naïve ICOS^−^-Tresp pool was detected (Figure 5A,B). Significant correlations between the percentage of RTE-Tresps within naïve Tresps and the percentages of Ki67^+^ cells within CD31^+^-memory Tresps and MN-Tresps could not be ascertained, proposing that the normal age-dependent differentiation of the RTE-Tresps ceased (Figure 5C–E,G, pathway 1 and 2). Merely, an age-dependent differentiation of resting-MN-Tresps (Figure 5A,B) into CD31^−^-memory-Tresps was still recognizable (Figure 5J,K). Irrespective of age, an increased, although not significant differentiation of resting MN-Tresps into CD31^−^-memory-Tresps was observed (Figure 5H,I). In summary, it seems that an age-dependent as well as an age-independent exhaustion of the differentiation of RTE-Tresps via CD31^+^-memory-Tresps and direct proliferation (Figure 5G, pathway 1 and 2) causes their enhanced differentiation via resting MN-Tresps, which are still converting rather than already proliferating into CD31^−^-memory-Tresps (Figure 5G, pathway 3). Consequently, in these patients, a further significant decrease of total ICOS^−^-Tresps within total CD4^+^-T-helper cells was observed, compared to those in remission and compared to healthy controls (Figure 5L). 

### 2.6. The Ratio of ICOS^+^-Tregs/ICOS^+^-Tresps of CD4^+^-Cells Is Significantly Reduced in Active SLE Patients

**Figure 6:**
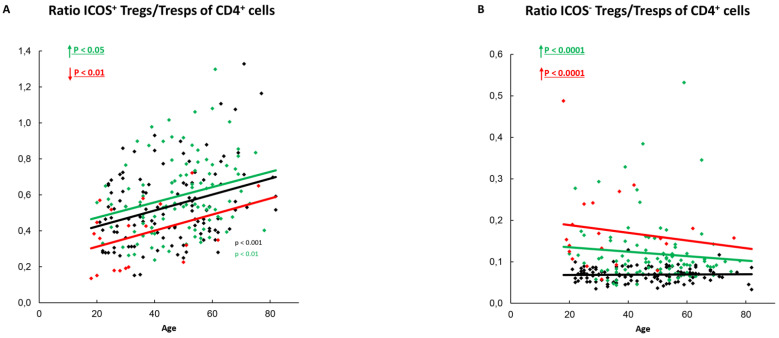
Ratio of ICOS^+^-Tregs/Tresps or ICOS^−^-Tregs/Tresps within total CD4^+^-T-helper cells. The ratio of ICOS^+^-Tregs/Tresps (**A**) as well as the ratio of ICOS^−^-Tregs/Tresps (**B**) is shown for healthy controls (Black coulored), SLE patients in remission (Green coloured) and SLE patients with active disease (Red Coloured). The figures present the regression lines concerning the changes with age. Significant age-dependent changes are marked by black, green, or red P-values. Significant age-independent differences between healthy volunteers and SLE patients in remission or SLE patients with active disease are marked by green or red underlined P-values. Further information of the calculated P-values is illustrated in Table S1.

To investigate whether the described changes in the differentiation of the different Treg/Tresp subsets can influence the ratio of ICOS^+^-Tregs/ICOS^+^-Tresps or ICOS^−^-Tregs/ICOS^−^-Tresps, we calculated these ratios for all patient groups as a function of age. In healthy controls, ICOS^+^-Tregs increased with age (Figure 2L), while ICOS^+^-Tresps remained unchanged (Figure 3L). This significantly increased the ratio of ICOS^+^-Tregs/ICOS^+^-Tresps with age (Figure 6A). In contrast the percentages of ICOS^−^-Tregs (Figure 4L) and ICOS^−^-Tresps (Figure 5L) did not change with age, so that their ratio also remained unchanged with age (Figure 6B). 

In SLE patients in remission, ICOS^+^-Tregs remained unchanged with age (Figure 2L), while ICOS^+^-Tresps decreased (Figure 3L), causing a significant increase in the ratio of ICOS^+^-Tregs/ICOS^+^-Tresps with age (Figure 6A). However, independent of age, this ratio was significantly elevated in these patients compared to healthy controls (Figure 6A). Age-related changes of ICOS^−^-Tregs (Figure 4L) or ICOS^−^-Tresps (Figure 5L) were not detected. Nevertheless, similarly to the ratio of ICOS^+^-Tregs/ICOS^+^-Tresps, the ratio of ICOS^−^-Tregs/ICOS^−^-Tresps was age-independently significantly increased (Figure 6B).

In SLE patients with active disease, age-related changes were neither detected for ICOS^+^-Tregs (Figure 2L) nor for ICOS^+^-Tresps (Figure 3L) and therefore also not for their ratio (Figure 6A). However, independent of age, this ratio was significantly decreased in these patients compared to healthy controls (Figure 6A). Age-related changes were also not detected for ICOS^−^-Tregs or ICOS^−^-Tresps (Figure 4L and Figure 5L). However, independent of age, the ratio of ICOS^−^-Tregs/ICOS^−^-Tresps was significantly increased in these patients compared to healthy controls (Figure 6B). 

## 3. Discussion

It is now known that abnormal T-cell activation in conjunction with aberrant T-cell signaling and cytokine production significantly impairs the function and differentiation of these cells and is thus crucially involved in the development of SLE [27]. Regarding CD4^+^-T-cells an accumulation of terminally differentiated effector memory T-cells with decreased proliferative capacity and increased apoptosis sensitivity was observed [28,29,30,31]. Consequently, a reduction in circulating CD4^+^-T-cells was documented [15,21,32]. As both immunosuppressive CD4^+^-Tregs and stimulating CD4^+^-Tresps are involved [13,15,33,34], it seems likely that a misguided differentiation of either CD4^+^-Tregs or CD4^+^-Tresps or probably of both CD4^+^-T-cell subsets causes an imbalance in the ratio of CD4^+^-Tregs to CD4^+^-Tresps in these patients. This balance has been intensively studied in recent years, providing contradictory results, and was shown to depend on the selection of the respective Treg/Tresp subsets [35,36,37,38,39]. 

In this study, we examined the differentiation of highly proliferative ICOS^+^- as well as less proliferative ICOS^−^-Treg/Tresp subsets in SLE patients in remission or active disease compared to healthy volunteers. By correlating the percentages of RTE-Tregs/Tresps within the naïve Treg/Tresp pool with their own Ki67 expression, as well as with that of resting MN-Tregs/Tresps, CD31^+^- and CD31^−^-memory Tregs/Tresps we identified three different differentiation pathways of RTE-Tregs/Tresps, first via CD31^+^-memory-Tregs/Tresps (pathway 1) and second via their direct proliferation into CD31^−^-memory-Tregs/Tresps (pathway 2). The additional differentiation of resting MN-Tregs/Tresps into CD31^−^-memory-Tregs/Tresps (pathway 3) was examined by correlating the percentage of resting MN-Tregs/Tresps within total CD31^−^-Tregs/Tresps with their own Ki67 expression, as well as with that of CD31^−^-memory-Tregs7Tresps. Our investigations revealed that in healthy controls a regular combination of these three pathways ensured a stable CD4^+^-Treg/Tresp cell homeostasis with an increasing ratio of ICOS^+^-Tregs/ICOS^+^-Tresps and a consistent ratio of ICOS^−^-Tregs/ICOS^−^-Tresps with age. Presumably, the increasing ICOS^+^-Treg/ICOS^+^-Tresp ratio contributes to the maintenance of self-tolerance with age. Recently, we showed in different pathologic conditions, such as preterm labor, renal insufficiency, and patients on renal replacement therapy, the described ratios to have a decisive role in maintaining proper Treg- and Tresp-cell functioning [40,41].

In SLE remission patients, the immunosuppressive therapy suppressed exaggerated differentiation of all these T-cell subsets substantially. However, it became apparent that despite a highly sophisticated immunosuppression, an increased additional consumption of resting naïve MN-Tregs/Tresps of these T-cell subsets was detectable. This phenomenon has been known for almost 20 years [28]. Yet, until today neither the T-cell subsets involved, nor the cellular mechanisms, causing transition from remission to active disease are known. Our data show that in these patients, remission is associated with an age-independent increase in the ICOS^+^-Tregs/ICOS^+^-Tresps ratio, which simultaneously increases in an age-dependent manner. Obviously, this was achieved by an age-independent increase in the conversion of resting ICOS^+^-MN-Tregs/Tresps, where the differentiation of resting ICOS^+^-MN Tresps changed from proliferation in healthy controls to conversion in SLE remission patients. In addition, we observed an age-dependent proliferation of ICOS^+^-RTE-Tregs into ICOS^+^-CD31^−^-memory Treg, which could not be assured for ICOS^+^-RTE-Tresps. An age-independently increased conversion of resting MN-T-cells into CD31^−^-memory-T-cells was also observed for both ICOS^−^-Tregs and ICOS^−^-Tresps, significantly increasing the ICOS^−^-Tregs/Tresps ratio. 

With active disease, the age-independently increased ratio of ICOS^+^-Tregs/ICOS^+^-Tresps broke down and was significantly decreased, while that of ICOS^−^-Tregs/ICOS^−^-Tresps continued to rise significantly, compared to that of healthy controls. In this regard, our data showed an exhaustion of RTE-Treg/Tresp differentiation in all Treg/Tresp T-cell subsets, with an age-independently increased differentiation of resting MN-Tregs/Tresps into CD31^−^-memory Tregs/Tresps appearing to be most decisively involved in the transition from remission to active disease. Here, the drop in the ratio of ICOS^+^-Tregs/ICOS^+^-Tresps seemed to be because differentiation of resting ICOS^+^-MN Tresps switched from conversion back to proliferation upon breakthrough of the immunosuppressive therapy, increasing the ICOS^+^-Tresp pool. In these patients, age-dependent differentiation of ICOS^+^-RTE-Tregs/Tresps via resting MN-Tregs/Tresps was no longer possible, but was replaced by pathways 1 and 2 for ICOS^+^-RTE-Tresps, but only in a limited way for ICOS^+^-Tregs. In contrast, the age-independent increase in the ratio of ICOS^−^-Tregs/ICOS^−^-Tresps was found to be since the differentiation of ICOS^−^-resting MN-Tregs changed from conversion to proliferation upon breakthrough of the immunosuppressive therapy, increasing the ICOS^−^-Treg pool. In these patients, age-dependent differentiation of ICOS^−^-RTE-Tregs/Tresps via resting MN-Tregs/Tresps was maintained, demonstrating that the differentiation of resting ICOS^−^-MN-Tregs/Tresps represents the final pathway by which CD31^−^-memory Tregs/Tresps are produced. However, although this final proliferation of the resting ICOS^−^-MN Tregs can significantly increase the total Treg pool, it may have a weakening effect on the functionality of the total Treg cell pool [21], as highly activated ICOS^−^-CD31^−^-memory-Tregs with strong proliferative capacity and high apoptosis sensitivity may arise and thus can hardly be re-stimulated. On the other hand, the inability of resting ICOS^−^-MN-Tresps to proliferate into ICOS^−^-CD31^−^-memory Tresps, resulting in ICOS^−^-CD31^−^-memory Tresps with lower proliferative capacity and apoptosis sensitivity may be responsible for the increased resistance of these cells for Treg suppression [19,21]. Consequently, our data show for the first time that conversion or proliferation of resting MN-Tregs/Tresps may influence both the number and the function of the arising memory-Tregs/Tresps. As different Treg/Tresp subsets such as ICOS^+^- and ICOS^−^-Tregs/Tresps are differently affected by these mechanisms, the immune homeostasis may be characteristically changed, as shown for SLE patients in remission or active disease. 

However, regarding the question of which specific ICOS^+^- or ICOS^−^-Treg/Tresp subsets are specifically involved in the pathogenesis of SLE, further investigations for the identification of characteristic markers on both ICOS^+^- and ICOS^−^-Tregs/Tresps may be necessary. It is known that ICOS is predominantly expressed on both follicular and regulatory follicular T helper cells (Tfh and Tfr cells) which regulate antagonistically the quantity and quality of humoral immunity [22,42]. Activation of this molecule was shown to induce Bcl-6-dependent functional differentiation of both cell subsets [43]. However, both increased and decreased ratios of Tfh/Tfr cells have been found to correlate with autoantibodies and disease activity in SLE patients, questioning the importance of this ratio [44,45]. Although in our study, ICOS^+^-Tregs/Tresps were not further characterized by Tfr or Tfh specific markers, such as BCl6, CXCR5 or PD-1, our data also suggest that the dual role of ICOS in both Treg and Tresp expansion, may contribute decisively to sensitive changes in their ratio in active disease patients. Thereby, our data reveal that excessive differentiation of thymus-derived ICOS^+^- or ICOS^−^-RTE-Tregs/Tresps and of the resulting mature naïve Tregs/Tresps affects significantly the ICOS^+^-Treg/Tresp or ICOS^−^-Treg/Tresp ratios. Differential expression of ICOS within the thymus was already shown for Tregs [23]. Therefore, it seems likely that such thymic-derived, naturally occurring ICOS^+^ or ICOS^−^-Tregs may be expanded in active SLE in order to compensate for autoreactive effector responses, which were shown for FoxP3^+^Helios^+^ Tregs and more recently for a special CD25^low+^FoxP3^+^ Helios^+^-Treg subset [46,47,48]. 

Nevertheless, it remains elusive which ICOS^+^- or ICOS^−^-Tresp subsets would be suitable to deliver meaningful results in such ratios in combination with ICOS^+^- or ICOS^−^-Tregs. Beside germinal center Tfh cells, various circulating Tfh-like populations and peripheral helper T (Tph) cells, expressing ICOS and producing IL-21, are now described. These cell populations resemble phenotypically Tfh cells but provoke autoantibody production outside of germinal centers [49]. Regarding ICOS^−^-Tresps which represent the largest subset within CD4^+^-T-helper cells, we observed a strong decline of these cells within total CD4^+^-T-helper cells in association with disease activity. These findings are to some extent consistent with previous studies describing lymphopenia as a common clinical feature and diagnostic criterion for SLE patients [50]. The underlying mechanisms of this phenomenon are still unclear. Increased apoptosis of peripheral T cells has long been discussed [51], where recent studies revealed an association between low expression of serine arginine-rich splicing factor 1 (SRSF1) and anti-apoptotic Bcl-xL gene in T cells and lymphopenia in patients with SLE [52]. However, these studies do not discriminate between T cell subsets such as CD4^+^- and CD8^+^-T-cells, or Tresps and Tregs. It should be noted that the Treg fraction of the diminished CD4^+^-T-helper cell pool is increased in SLE patients, whereas the Tresp fraction is decreased. Therefore, these findings presumably rather apply for Tregs than for Tresps. Our findings, showing proliferation of ICOS^−^-resting MN-Tregs but conversion of ICOS^−^-resting MN-Tresps rather suggest the formation of apoptosis-sensitive ICOS^−^-Tregs with diminished functionality, but apoptosis-resistant ICOS^−^-Tresps with reduced sensitivity for Treg suppression. In summary, our data show strong exhaustion of individual differentiation pathways of RTE-Tregs/Tresps in SLE patients with active disease, where final differentiation via proliferation or conversion of resting ICOS^−^-MN-Tregs/Tresps may provoke mainly functional changes of the arising ICOS^−^-memory Tregs/Tresps. In contrast, similar mechanisms may cause quantitative changes of ICOS^+^-Tregs/Tresps causing disease flares in SLE remission patients. Thereby, the generally restricted differentiation capacity of ICOS^+^-RTE-Tregs via all three pathways was found to represent a reliable marker for the discrimination of SLE patients in remission or active disease. Hence, immunosuppressive medication targeting ICOS^+^-Tresp differentiation in SLE patients while enabling Treg differentiation could be a goal of further investigations. Our calculations and the resulting interpretations may have limitations such as unequal numbers of subjects in the groups and that our strategy may be further confirmed. Meanwhile, further studies suggest accelerated immune senescence, not only for CD4^+^-T-cells, but also for other immune cells, such as CD8^+^-T-cells and B-cells, as well as for cells of the innate immune system, proposing similarities in immune dysfunctions between SLE patients and elderly people with advanced age [53]. Moreover, from clinical point of view, gender specific differences should be expected which need further attention in following studies.

## 4. Materials and Methods

### 4.1. Patient Collectives and Healthy Controls

Peripheral blood samples were collected from 115 healthy controls (Group 1) and 116 SLE patients. All SLE patients fulfilled the 1983 revised and 1997 updated criteria of the American College of Rheumatology (ACR) for SLE [54,55] and were diagnosed as in remission (SLEDAI ≤ 7, Group 2, *n* = 96) or suffering from active disease (SLEDAI > 7, Group 3, *n* = 20). Of all SLE patients, 89 (77%) showed kidney involvement. Blood samples were collected during routine visits or during a hospital stay at the Department of Nephrology, University of Heidelberg. 

### 4.2. Positive Selection of CD4^+^-T-Cells

Peripheral venous blood samples (9 mL) were collected from all participants into EDTA-containing tubes. CD4^+^-T-cells were isolated by immune affinity chromatography with the FABian system (IBA GmbH, Göttingen, Germany), according to the manufacturer’s instructions. This cell isolation process is performed in the FABian column prefilled with a Strep-Tactin-Fab-anti-CD4 coated agarose matrix. The process starts with sucking up whole blood samples. CD4^+^-T-cells bind to the matrix, while non-target cells are washed away. Adding D-biotin to the matrix causes a dissociation of Fab and target cells from the beads so the CD4^+^-T-cells can be recovered. Subsequently the isolated CD4^+^-T-cells are analyzed using six-color flow cytometry.

### 4.3. Fluorescence-Activated Cell Sorting (FACS) Staining

Briefly, at most 8 × 10^6^ CD4^+^-T-cells were surface stained with 5 µL peridinin-chlorophyll-protein-Cy5-5 (PerCpCy5.5)-conjugated anti-CD127 (eBioscience, Frankfurt, Germany), 20 µL phycoerythrin (PE)-conjugated anti-ICOS (BD Biosciences, Heidelberg, Germany), 5µL allophycocyanin-H7 (APC-H7)-conjugated anti-CD45RA (BD Biosciences), and 5 µL phycoerythrin-cyanine 7 (PE-Cy7)-conjugated anti-CD31 (eBioscience) mouse monoclonal antibodies. Intracellular staining for the detection of FoxP3 was performed using a fluoresceinisothiocyanat (FITC)-conjugated anti-human FoxP3 staining set (clone PCH101, eBioscience) according to the manufacturer’s instructions. Detection of Ki67^+^ cells within the different Treg/Tresp subsets was performed by incubating the fixed cells with 2 µL Alexa-flour 647-conjugated anti-Ki67-conjugated mouse monoclonal antibodies (clone B56, BD Biosciences). Negative control samples were incubated with isotype-matched antibodies. FSC-H versus FSC-A and FSC versus SSC gating was used for doublet and debris discrimination (for gating strategy see Figure S1). Cells were analyzed by a FACS Canto flow cytometer (BD Biosciences). Statistical analysis was based on at least 100,000 CD4^+^-T-cells.

### 4.4. Statistical Analysis 

Linear regression was used to evaluate the influence of age on the composition of total ICOS^+^- or ICOS^−^-Treg/Tresp cell pools with their subsets (RTE-, MN-, CD31^+^-memory- and CD31^−^-memory-Tregs/Tresps) for healthy controls, SLE remission patients, and patients with active disease using separate models. The same approach was used for evaluating the changes with age in the composition of the naïve ICOS^+^- or ICOS^−^-Treg/Tresp cell pools with RTE- and MN-Tregs/Tresps, as well as the changes with age in the composition of the ICOS^+^-or ICOS^−^CD31^−^-memory Treg/Tresp cell pools. In addition, we calculated the Pearson correlation coefficients (r) between age and the change of variables. A P-value < 0.05 was considered significant.

In order to discover the differentiation pathway of ICOS^+^- or ICOS^−^-RTE-Tregs/Tresps via CD31^+^-memory-Tregs/Tresps or proliferation via MN-Tregs/Tresps, an analogous procedure was chosen by correlating the changes in the percentages of ICOS^+^- or ICOS^−^-RTE-Tregs/Tresp within their naïve CD45RA^+^-Tregs/Tresps with the Ki67 expression of their respective RTE-, MN, CD31^+^-memory, and C31^−^-memory Tregs/Tresps. Similarly, in order to examine the differentiation of resting ICOS^+^- or ICOS^−^-MN-Tregs/Tresps into ICOS^+^- or ICOS^−^CD31^−^-memory Tregs/Tresps, the changes in the percentages of MN-Tregs/Tresps within CD31^−^-Tregs/Tresps were correlated with the Ki67 expression of their respective MN- and CD31^−^-memory-Tregs/Tresps. A P-value of < 0.05 was considered significant. Age-independent differences concerning the above listed Treg/Tresp subsets between healthy volunteers, active SLE patients, and inactive SLE patients, were examined using multiple regression analysis adjusted for the age variable (centered on the mean), wherein an interaction term of the age and the patient group was included. Software package BiAS for Windows (version 10.06) was used for all tests.

## Figures and Tables

**Table 1:** Clinical characteristics of SLE patients and healthy controls.

	Healthy Controls	SLE Remission Patients	Active SLE Patients
	*n* = 115	*n* = 96	*n* = 20
Female Sex, *n* (%)	92 (80%)	77 (80%)	16 (80%)
Age (years)	46 (21–82)	51 (20–78)	31 (18–76)
Time since initial diagnosis (months)		192 (2–563)	19 (0–328)
Renal involvement, *n* (%)		73 (79 %)	16 (80%)
SLEDAI		0 (0–10)	13 (6–24)
ANA titer ≥ 1:1280, *n* (%)		37 (40%)	10 (50%)
DsDNA antibodies ELISA (IU/mL)		20 (3–1051)	134 (26–708)
C3 complement (g/L)		1.2 (1.0–1.6)	0.7 (0.2–1.0)
Erythrocyturia (× μL^−1^)		2 (0–1038)	34 (1–1027)
Leukocyturia (× μL^−1^)		3 (0–634)	38 (0–224)
Serum leucocytes (× nL^−1^)		6 (3–13)	4 (3–16)
Serum creatinine (mg dL^−1^)		0.8 (0.5–5.3)	0.8 (0.5–5.3)
CKD-EPI GFR (mL min^−1^ (1.73 m^2^)^−1^)		96 (11–140)	101 (10–138)
Urine-Protein/Urine-creatinine Ratio (g (mol creatinine)^−1^)		11 (4–468)	165 (6–727)
Medication			
No Medication, *n* (%)		8 (8%)	2 (10%)
Antimalarials, *n* (%)		72 (75%)	15 (75%)
Mycophenolic acid (MPA), *n (*%)		41 (43%)	8 (40%)
Azathioprine (AZA), *n* (%)		18 (19%)	2 (10%)
Glucocorticoids, *n* (%)		41 (43%)	14 (70%)
Glucocorticoid dose (mg d^−1^)		2.5 (0–4)	4 (0–40)

Abbreviations: ANA, antinuclear antibodies; CKD-EPI GFR, Chronic Kidney Disease Epidemiology Collaboration estimated Glomerular Filtration Rate; MDRD GFR, Modification of Diet in Renal Disease Study estimated Glomerular Filtration Rate; SLEDAI, SLE disease activity index; *n*, number. The data is presented as median (range) or number (percent).

## Data Availability

Original data on flow cytometric determinations are available upon request.

## Supplementary Materials

The following are available online at https://www.mdpi.com/article/10.3390/ijms22179501/s1.
