# Supplementary material for: Impaired Differentiation of Highly Proliferative ICOS+-Tregs Is Involved in the Transition from Low to High Disease Activity in Systemic Lupus Erythematosus (SLE) Patients
Source: Int J Mol Sci. 2021 Aug 31;22(17):9501. doi: 10.3390/ijms22179501 (PMC8430608; doi:10.3390/ijms22179501)
Supplement: Supplementary file 1 [file ijms-22-09501-s001.zip › ijms-1254433-supplementary.pdf]

Table S1: Exemplary presentation of results of the statistical analysis.

P-value	Meaning	Statistical test	Exemplary statistical output
P	Correlation of T-cell subsets with age for healthy controls	**Linear Regression** (according to Pearson)	Fig 1A, x=age, y=RTE-Tregs of ICOS$^+$-Tregs<br>$p < 0.0001$ (R -0.41, β -0.04, t (113) = 4.85)
P	Correlation of T-cell subsets with age for SLE patients in remission		Fig 1A, x=age, y=RTE-Tregs of ICOS$^+$-Tregs<br>$p < 0.001$ (R -0.39, β -0.02, t (94) = 3.96)
P	Correlation of T-cell subsets with age for active SLE patients		Fig 1A, x=age, y=RTE-Tregs of ICOS$^+$-Tregs<br>$P < 0.05$ (R -0.47, β -0.15, t (18) = 2.23)
P	Age independent differences between SLE patients in remission versus healthy controls	**Multiple Regression** (adjusted for age, centered on the mean)	Fig 1A, x=age, y=RTE-Tregs of ICOS$^+$-Tregs<br>↓$P < 0.0001$ (R=0.47, β -0.64, $f^2 = 0.29$)
P	Age independent differences between active SLE patients versus healthy controls		Fig 1A, x=age, y=RTE-Tregs of ICOS$^+$-Tregs<br>↑$P < 0.001$ (R=0.52, β 1.06, $f^2 = 0.38$)
Δp	Differences in the slope of the regression lines between T-cell subsets of SLE patients in remission and healthy controls	**Comparison of regression lines**	Fig 3C, x= Ki67$^+$-cells of ICOS$^+$-RTE-Tresps,<br>y= RTE-Tresps of ICOS$^+$-CD45RA$^+$-Tresps (%)<br>$\Delta p < 0.01$
Δp	Differences in the slope of the regression lines between T-cell subsets of active SLE patients and healthy controls		Fig 2C, x= Ki67$^+$-cells of ICOS$^+$-RTE-Tregs,<br>y= RTE-Tregs of ICOS$^+$-CD45RA$^+$-Tregs (%)<br>$\Delta p < 0.0001$

Suppl. Fig. 1

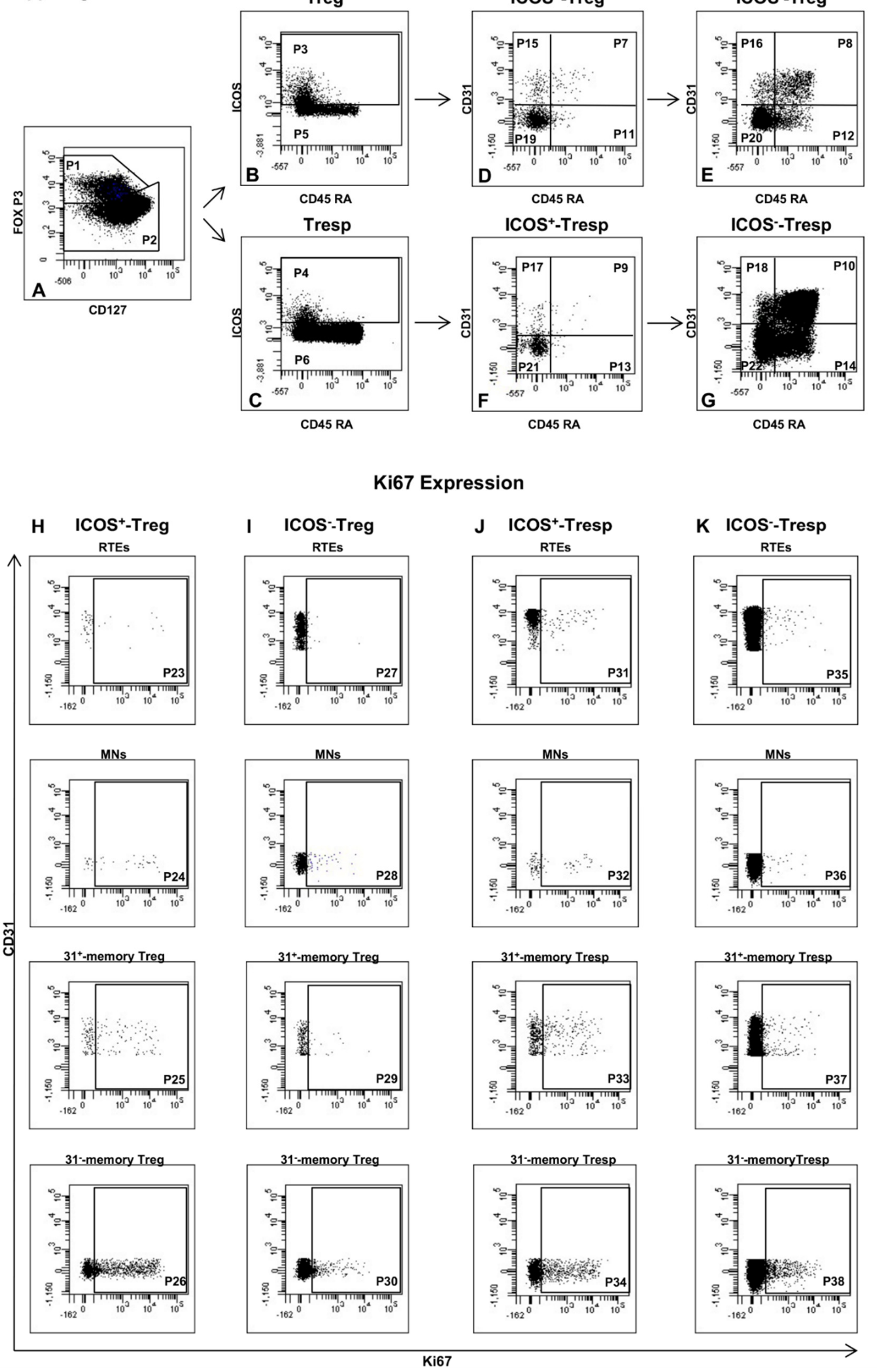

Scheme S1. Gating Strategy. Positively isolated CD4$^+$-T-helper cells were divided into CD4$^+$CD127$^{low+/-}$FoxP3$^+$-Tregs (P1) and CD4$^+$CD127$^+$FoxP3$^-$-Tresps (P2) by gating fluorescence intensity of FoxP3 versus CD127 (A). ICOS$^+$-Tregs/Tresps (P3, P4) and ICOS$^-$-Tregs/Tresps (P5, P6) were gated by fluorescence activity of ICOS versus CD45RA (B and C). The percentages of ICOS$^+$- and ICOS$^-$-RTE-Tregs/Tresps (P7, P8, P9, P10), MN-Tregs/Tresps (P11, P12, P13, P14), CD31$^+$-memory Tregs/Tresps

(P15, P16, P 17, P18) and CD31^{-}-memory Tregs/Tresps (P19, P20, P21, P22) were estimated by analyzing ICOS^{+}-Tregs (P3), (D), ICOS^{-}-Tregs (P5), (E), ICOS^{+}-Tresps (P4), (F) and ICOS^{-}-Tresps (P6), (G) for its fluorescence intensity of CD31 versus CD45RA. The Ki67 expression of ICOS^{+}- or ICOS^{-}-RTE, MN, CD31^{+}-memory-Tregs and CD31^{-}-memory-Tregs (P23-P26 and P27-P30), as well as Ki67 expression of ICOS^{+}- or ICOS^{-}-RTE, MN, CD31^{+}-memory-Tresps and CD31^{-}-memory-Tresps (P31-P34 and P35-P38) were estimated by analyzing the fluorescence intensity of CD31 versus Ki67 (H-K).
